# Whether Disabled Parents Receive Personal Assistance for Parenting and the Consequences for Children—An Interview Study

**DOI:** 10.3390/ijerph19063330

**Published:** 2022-03-11

**Authors:** Ulrika Järkestig Berggren, Ann-Sofie Bergman

**Affiliations:** 1 Department of Social Work, Linnaeus University, 39231 Kalmar, Sweden; 2 Department of Social Work, Stockholm University, 10691 Stockholm, Sweden; ann-sofie.bergman@socarb.su.se

**Keywords:** children, parent, personal assistance, disability, young carers

## Abstract

Personal assistance, since its implementation in 1993, has been shown to provide support for persons with severe functional disabilities in their everyday life, ensuring inclusion in societal roles such as working life. Personal assistance (PA) may also provide support in parenting; however, with the right to PA becoming increasingly questioned in Sweden, parents with disabilities have varying experiences of receiving support for their role as parents. Experiences also differ in regard to how access to a personal assistant is important to their child’s daily life. The aim of this article is to shed light on the meaning of PA for parents and children in everyday life, especially when PA is reduced or even withdrawn. Eleven parents who have had or presently have PA were interviewed. The results show that parents describe that PA help them to fullfil their parental roles although the support could be more flexible to the needs of parents and their children. In situations when PA has been denied, children are negatively impacted and some children act by taking on responsibilities for the care of their parent. In conclusion; childrens’ perspective of their family life needs to be taken in consideration when assessing the rights to PA.

## 1. Introduction

The right to live a life of equal participation, meaning being part of all the different arenas in society such as being a parent, has been stated in national law in Sweden as well as in the UN Convention on the Rights of Persons with Disabilities [1,2]. In Sweden, the implementation of the “LSS” Law regulating Support and Service to Persons with Certain Functional Disabilities (1993, p. 387) aimed to provide support for equal living conditions and participation for persons with disabilities. LSS presented Personal Assistance (PA) as the most novel support form in LSS when it was implemented in 1993, after inspiration from the independent living movement in the USA. Studies have repeatedly shown that PA provides support that secures inclusion in everyday life, although the research has given fairly little attention to support of the role as parent [3,4,5].

Since 2014, however, the social policy ideology of equal participation that is the backbone of LSS is being replaced with a government policy of implementing austerity measures on the granting of PA and initiating a commission (dir 2016:40) of changes in the LSS legislation with the explicit aim to cut costs [6,7]. After criticism in the 2018 election year, this objective was modified with the aim to keep the current cost level. The proposed bill (in 2021) addresses some of the points of the criticism, such as PA for children given that there is a difference between a parent’s normal responsibility and support that should be given by PA [8]. The perspective of parents who use PA in their parenting has however not been addressed in any of the recent propositions. The most recent bill does not even mention ‘parent’ as a role for persons with disabilities, let alone mention children of parents who have disabilities [9].

The number of persons who receive PA has decreased from 16,000 in 2015 to 14,000 in 2019 (SSIA, 2020). However, there have been no changes in the law, meaning that all needs assessments must still be made according to the existing legal framework. 

The PA included within LSS is to ensure support in basic needs such as personal hygiene, meals and communication, which are held separate from additional needs such as parenting. In order to be eligible for assistance compensation from the SSIA, basic needs must be at least 20 h a week. This means that to have support in parenting you must have basic needs for 20 h a week, and only then will your needs for support in parenting be assessed and possibly granted. The municipality grants assistance needs up to 20 h a week. The bill preceding the law stated that it is important that parents with disabilities are given support in order to secure that they can fulfill their role as parents; however, PA is only described as a support for the youngest children to ensure that their needs are met by a small number of persons [10]. It is further stated that when parents need other forms of support, the responsibility falls to the social services. A study of the assessment process during 2013–2017 for PA revealed first that few parents who apply for PA are granted PA [11] and second that parenthood is rarely considered in the assessment [12]. 

In 2020, the United Nations Convention on the Rights of the Child became law in Sweden, emphasizing among other rights the right of the child to have a family (article 7). This underscores the importance of viewing PA as a support not only for the adult person in the role as parent, but also for the child who has a right to be with her/his family. The law LSS is now 29 years old, and social policy has shifted, leaning towards economical reasoning (rather than rights for persons with disabilities) as well as towards a strengthened view on children’s rights in society. 

The aim of this study is to shed light on how PA provide support in parenting, and how children and parents are affected when PA is reduced or even withdrawn. 

### 1.1. Research on Parenting with Personal Assistance

The societal discourses about disability do not include parenthood as a role for disabled adults. From the parents’ perspective, there are experiences of demeaning attitudes and having their identity of being a parent questioned by persons in authority as well as the general public who do not acknowledge them as care givers and partners [13,14,15]. 

The bulk of research on parenthood among persons with disabilities has studied motherhood rather than fatherhood [16,17]. Although the discourse of what is regarded as ‘a real woman’ includes motherhood in our culture, mothers with disabilities are embedded in an overarching discourse of disability and have difficulties being perceived as women or mothers [17,18]. Grue and Tafjord Lærum [18] show that mothers experience that other people react to their motherhood and that they are more closely monitored in their role as a parent. They feel that they have to work hard in order to convince other people that they are capable and good enough parents. The studies on mothers show that mothers clearly separate emotional care, such as comforting the child, and rule-setting that are both considered as exclusively the parents’ responsibility, from practical care such as changing diapers that the personal assistant can provide [17,18,19].

The limited studies about fatherhood show that there are differing views on fatherhood. Some men experienced that taking on caregiving tasks for their children is meaningful, while others had problems in accepting a task that they did not consider to be masculine and losing the role as the family’s breadwinner could affect their self-esteem [20]. The traditional view on motherhood and fatherhood places the responsibility for care on the mother, and activities and play on the father [14,17]. A more modern understanding of parenthood emphasizes the sharing of responsibilities for care of the children regardless of gender [21]. Selander and Engwall [17] conducted a follow-up study after ten years, looking at parenting with PA and showing that the parents have kept their parenting strategies. The interviews also showed that over the years the parents had processed their feelings of sadness and frustration of not being able to carry out some parenting tasks, and today they could accept and appreciate that PA could fill in for them and that through PA they could provide their children access to things and experiences.

A theme in research concerning children of parents with disabilities describes the situation where children take on responsibilities to care for their parents and become young carers. The view of children being young carers differs among countries. In England, research and legislation show acceptance that children do take on these tasks, and children over 16 can get a carer’s allowance (NHS, 2022) for performing caring tasks [22]. In Sweden, children taking on caring responsibilities is considered a potential problem that should be avoided [23]. Furthermore, the view on children of parents with disabilities is contested as to the extent, the seriousness and the causes of how the disability affects the family, whether connected to the discourse of an imperfect parenting or rather as a consequence of lack of support from society in some parenting situations [12,13,15,24,25].

Bergman, Emilsson and Järkestig Berggren [12] found that parents applied for PA for practical support, care and supervision, emotional support and support with communication, and support to be involved in their children’s lives. Few parents did receive PA for parenting, partly due probably to the construction of the LSS law and partly due to fear that applying for support in parenting may lead to being viewed as an imperfect parent. Regardless of gender, both mothers and fathers expressed fear that their children can be taken away from them if they are viewed unfit as parents. 

### 1.2. Rights for Parents and Children in a Disability Framework

The study uses a relational perspective on disability that means that disability is a relationship between the individual and the society [26]. Disability occurs when there is a “poor fit” between the individual’s capabilities which are not within the typical range of the society and the demands of the societal environment which are not adapted to the whole range of society. A person is then defined as disabled if a limitation, disease or impairment causes him or her to experience significant barriers in everyday life. Furthermore, whether a specific impairment is disabling or not depends on the situation or context [27]. In connection with parents and children, this study uses a right’s perspective of parents and children. What then is a human right? According to Carreira da Silva“a right is a mutual relation, an institution made of political claims involving at least two individuals. A right refers to entitlements, liberties, powers or immunities that have been codified in international covenants and declarations, as well as in national constitutions.” [28]. Human rights are not given a priori, but in da Silva’s understanding they uphold in a relation. Building on this, we turn to the UN Convention on the Rights of Persons with Disabilities (CRPD), which is ratified by Sweden, and in article 23 the right to marriage, to family life and to be a parent are granted.

Then turning to the UN Convention on the Rights of the Child (CRC), made law in Sweden in 2020, the CRC applies the perspective of children as agents with the capacity and right to act on their own behalf in questions that matter to the child. Also, the CRC states that authorities are always to consider what is in the best interest of the child in every decision. Regarding family, article 2 states that children are to be protected against discrimination on the grounds of parents’ disabilities. Furthermore, article 7 also grants children the right “to know and be cared for by his or her parents”. 

Taken together, the UN Conventions from a family perspective grant the right to their family for both parents and children. This family perspective has been criticized for hiding that the parents’ and children’s perspectives and needs might differ or even contradict one another at times. This conflict in perspective may occur in any family, but it can easily fall into the strong societal discourse of failing parenthood that adult persons with disabilities face [13,14,15]. One way of handling the rights for children and parents alike may be to turn to “the best interest of the child” called for in the CRC, as the value measure for every act and decision. According to Lappeteläinen, Sevón & Vehkakoski viewing parental strategies in light of the best interest of the child may show that successful parenting can use several strategies [13]. 

## 2. Materials and Methods

This article presents the parental perspective about parenting with PA. The interviews with parents is part of a larger research project about parental support through PA, that also covers quantitative and qualitative data from applications and assessments of the right to PA in parenting as well as interviews with personal assistants. The study has been approved by the regional ethical review board in Linköping (ref. 2017/149-31). In exploring parents’ views on parenting with PA, we chose to do in-depth interviews with parents using a thematic scheme about parenting with PA, about everyday life with assistance and how they divided tasks especially in regard to parenting, and how they perceived that their children experienced the assistance. Hence, parents were given the chance to rather freely tell their stories of being a parent and about their children and the assistance. 

For this particular study, eleven parents were interviewed, six women and five men. The parents were recruited through assistance companies, through parent networks and through online communities. One parent had a grown-up child who had moved from home, and one parent had shared custody of a child living with her every other week. All other parents lived with their children full-time. The ages of the children ranged from 3 years of age up to 18 years. Ten of the parents currently had assistance from 16 h a week up till around the clock. One parent had lost the assistance altogether. Since all parents had PA at some point, they all have a severe physical impairment that affects their everyday life, thus meeting the definition of the basic right to PA. We have made a choice, however, not to focus on the medical diagnosis, and hence, this is not further described.

The interviews were conducted at the parent’s home, workplace, at the university, at the assistance company office, or at a library according to the parent’s own choice. All interviews lasted between 1–2 h, and they were recorded and transcribed verbatim after the interview. 

In analyzing the interviews, we initially coded for the meaning of assistance in parenthood using *parent, PA* and *support* as code words. Two overarching themes were found. The theme of using PA in parenting included the categories of the decision to use PA or not and the wish for more flexible assistance. The theme of the experience of losing and fearing losing the assistance included the categories, the consequences for the parent and for the child and what was lost in the absence of assistance. The process of analysis was conducted going back and forth between our themes and categories and the interview as a whole to make sure that we captured the overall meanings and that we did not detect any ambiguities [29].

## 3. Results

### 3.1. To Have or Not to Have Personal Assistance

All interviewed parents described what PA has meant for them not only in their lives as parents, but also in higher education, in working life and as partners. Christian had PA as a student, but lost his assistance in a re-assessment before he became a parent. In the interview, he speaks about the possibilities he gained through PA.

*I received PA and I am very happy for that. If I had not got that help during that time (while he was a student) I would not have graduated, and I would not be where I am today.… They should really broaden what you can get PA for. Look at it as an investment I had… I can say that had I not got the help in this situation, I would not have my own house and my work and so on… I have paid taxes now for many years, so I have probably re-paid that cost.*

*When Christian became a father, he no longer received PA due to more narrow assessment, so he talks about how his wife and at times his mother took care of his daughter in practical aspects when she was a toddler.*

*I take care of my own personal needs, but that is at the expense of other persons and of other things. I take care of my work, but not so much my home… What the state has won, you could say, has been sacrificed by someone else, and by me, too. I have had to adapt to the situation.*

Being a parent of a four-year-old, Christian talks about situations in his parenthood where he feels that he cannot perform what he describes as “normal” parenting. For example, dressing his daughter takes a lot more time.

*I have noticed that if I am to be a normal parent in this aspect then I need assistance, or I will be an abnormal parent in the time it takes to help her dress. When she was younger and there was a sweater and this and that, and I could not help at all. It took so long time. I started two hours early to be finished and still I did not succeed, so I called my parents and they came to help. That was tough.*

Christian further says that he may not be the parent who does the practical stuff, but he spends a lot of time with his daughter, and he is very affectionate and comforting with her. 

In talking about PA in parenthood, Christian expresses dual feelings; he says that with PA, some practical responsibilities that now rest on his partner could have been lessened. He also comments that a personal assistant helping out in day-to-day practical situations would have given himself more freedom in parenting, in taking his daughter out, for example. At the same time, Christian expresses a fear of letting new persons in, especially if there was to be a number of different people in their home. He wants to protect his daughter from him needing too many people in his life.

Christian says that he prefers to challenge himself, and when he needed help in the practical stuff with his parental responsibilities, he asked his family rather than have unknown professionals around. This desire to be a competent parent coping on his own is expressed also by other parents. The wish to have PA is not a decision taken lightly.

Cilla describes how she chose to employ her own sister when she got her first child. She was anxious about letting unknown people into her new role as a parent, because she felt vulnerable and insecure about being a parent for the first time.

*In the normal case, you know a new mom would want to be with her newborn in her own bubble with her family. And then to let someone in… I was afraid to allow someone in. Maybe an “expert” would say you should not do like that, and I would not be strong enough to take the discussions. So, I asked my sister (to be the assistant) and I was worried about that, too. It does alter our relationship. But it went well, and she stopped after the first year, and I felt safe enough to allow someone else in.*

Elisabeth who has a twelve-year-old daughter has used PA during the upbringing of her daughter, and she talks about having clear boundaries for what an assistant is allowed to do and about the relation to her daughter.

*I have always been crystal clear about that I am the mom and the assistants are not. They cannot step in at any point to take over… No, we are playing by my rules.*

Parents express that they take much consideration into whether they need help in their parenting role which for most parents challenges their self-confidence as a parent, as well as how they use the PA in parenting and still exercise their role as mother or father. Over time, the interviewed parents do seem to come to terms with asking for help, and they find PA a valuable support in their parenting.

### 3.2. Fear of Losing the PA Support

Although parents may have mixed feelings about accepting PA in their parenthood, the parents who have PA are concerned about being able to keep this support, and they all express fear at losing the PA in one of the recurrent assessments. 

Beatrice has three children; two teenagers live with her, and a 12-year-old lives with her part-time. She has had PA since before she had children. She has PA around the clock, and when the children came, she had awake PA hours all night to care for them, but now she fears that her night-time hours will be denied in her next assessment.

*We will see how long this will last, you never know nowadays. They cut down wherever they can, so… This fear is ever present because as things are for me now, to lose hours means that I cannot live the life I live now, quite simply.*

Celia uses a wheelchair and has a daughter. She has had PA for more than 20 years and has been through many re-assessments. She describes her feelings towards the periodic reviews:

*During all these years, I have had PA since 1995 I think, and during all these years I have been in fear of losing the PA … I took for granted I would get more time when I had my child, so I asked for a re-assessment myself. I thought my new parenthood would be a cause for more time in my PA. But I got a negative assessment to this request. They did not even understand my request.*

In the quote above, Celia points out the difficulty of getting hours specifically for parenting. As described earlier, to have hours for parenting, the applicant must have basic needs (such as personal hygiene, eating, etc.), and support in parenting falls under what is deemed as additional needs. Parents who apply for additional time in parenting have experiences of vastly differing assessments of their needs for time in parenting. Above Celia described her experience that her parental needs were not even understood in the first assessment. In a later re-assessment, she met another official who was to assess her application for support time in parenting.

*There came a new official and I was so scared that I would lose my assistance, but he told me to be calm. He would have to determine his decision with his manager, but he thought that it was not right that I had tried to raise my needs for assistance in parenting and no one had listened. So, he went on asking questions about my need for assistance as a parent. What I would like to do with my daughter. My daughter has a father, but I would like to be the mother in all ways, being a bit playful with my daughter, you know. Or do outings with her, accompany her to her leisure activities. I do not remember what the exact decision was, but I think it was two or three hours a week, doing an outing on weekends and accompanying her to her sport activity during the week. My daughter must have time for her activities; otherwise she would miss out on them, because I cannot go with her.*

Some of the parents have experienced losing their assistance altogether. John is divorced, and when his youngest son (12 years old) came to live with him full-time, John applied for extra time as a parent. The application decision came back withdrawing all assistance hours. From one day to the next, John had no assistance at all.

*The assistants were let go from one day to another. My old mom has not stopped crying in fear of the next time my assistance will be re-assessed. My son did not take this very good, and I did not feel well at all.*

John appealed to the court and won in the first instance; however, the authority appealed the case and lost there as well. The whole process took one year and eight months during which John and his son experienced insecurity with his assistance.

Both Celia and John express that access to PA also directly impacts their children’s lives. The next theme explores how parents view the consequences for their children.

### 3.3. Consequences for Children

In supporting parents, PA means possibilities and assets for their children. Parents express both what assistance means for their children and what are the consequences for them when there is a lack of PA.

One area that parents explicitly talk about is children’s activities. Parents who do not have assistance around the clock apply for specific assistance hours to be able to accompany their children to their activities. There are parents who experience that their hours have been cut, and then their child will miss out if there is not another parent or family member or friend who can take the child instead. 

Losing assistance is also an experience for some parents who after appealing got the right back to their assistance. In the meantime, both the parent and the child experienced anxiety over how the next day as well as the future would turn out. John describes how his son reacted when he suddenly lost the assistance:

*He started refusing to go to school because… in a way, it became hard for him at school, sitting and worrying how dad is coping. What will happen at home? No, I will stay at home.*

John’s son worried about him, and the anxiety of what can happen to his dad has stayed with him even though his dad has got his assistance back. John feels that the trauma of losing the assistance has affected both him and his son in a negative way, causing the problem of being distrustful that their life will be ok.

In John’s description, his son takes on responsibility for his father by not wanting to leave him alone, unsure of how his father will cope without anyone around. Other parents describe the same consequence when no assistant is available: the children feel anxious and take on responsibility for their parent. Anita, whose daughter is now in her early twenties, tells about a period in her life when she did not have PA, but had home care service, and she had to rely on her daughter for many practical things. “A six-year-old should not have to help her mother put on a jacket.”

Later on when Anita’s condition got worse, she did receive PA, but at one point she lost many hours in a re-assessment, which meant that her daughter had to take on responsibility for helping her. Anita tells about the situation:

*When I lost all those hours…it ended up with my daughter not bringing home friends to our house, because what if her mother needed her… like if her mother needed her for going to the toilet, like that. It would have been really embarrassing for her if she had friends over then.*

Anita expresses criticism that the lack of PA hours placed responsibility on her daughter that she should not have been forced to take on and imposed limitations on her daughter’s life. In any re-assessment, there has never been any consideration of the need she has had in her parental role.

Obviously, the lack of assistance hours poses difficulties for parents and has consequences also for children. Parents talk about strategies to try to save assistance hours to avoid being dependent on their children and to do schedules for their assistance that allow them to accommodate their children’s activities in school or during leisure time. When listening to Alexander and John, it becomes evident that having access to PA in their parental role also makes a difference in their children’s lives. Alexander and John are both fathers to teenagers who like to get together with friends and go out on weekends. Alexander has assistance around the clock, and he also has a car that he can drive assisted by his PA. John has limited hours in assistance and not in the evenings. He does not have a car, and he describes his experience with his teenage son:

I am that kind of sort. I don’t want my son to go somewhere if he can’t come home. I do need someone who can go with me to pick him up. Yes, it is these unplanned situations that happen. My son suddenly says he wants to go to town to meet his friends, and then he stays too long, and the bus has stopped going. Then there is not much I can do… It gives me stomach pain, I can tell you. I have no assistance in the evenings, so I can’t go get him.

Alexander, on the contrary, describes how he frequently drives his car to pick up his daughters in the evenings. He even got a gift from them, a keyring with a tag saying:

“Daddy’s car 24/7.” Alexander says that he is very happy to be able to support them in this way, and he feels good knowing that he can go to his daughters whenever needed. These two fathers talk about having the possibility to be flexible in their parental role. As mentioned, parents try to plan their assistance to accommodate their children, but when sudden situations occur such as going on outings with friends, several of the parents describe that they find it difficult to do these unforeseen activities, since hours are limited and applying for extra time takes several weeks.

The PA is further described to be inflexible in that it is only meant to be support for the person who receives the assistance, and the assistant cannot do anything if the parent is not taking part. Jonna who has a son in pre-school talks about her son missing out on going to birthday parties.

*An example, he is invited to a birthday party by a friend from kindergarten. There is no solution to this situation. Just imagining, I would most probably not be able to go with him. If I need to go to the toilet, I can’t get into the toilet. It would be much simpler if my son could be accompanied by my assistant alone. But that is not allowed. I must always be present with the assistant. If I need someone to go with my son, I have to apply to social services who will find someone that my son has never met and will not feel safe with, just to go to a party with his friends. I find this very odd.*

Jonna talks further about the restrictions she perceives to her possibilities of being a parent on the same conditions as any other parent. Being a parent also means to have her son’s friends over for lunch or dinner at times. According to the assignment for personal assistants, however, the assistant is not allowed to assist anyone other than the service user, and in her case also her young son since she lives alone. Therefore, the assistants are not allowed to cook for her son’s friends which means that she can’t have her son’s friends over and invite them for a lunch or dinner. Hence, she thinks that this has negative impact on her son’s social life.

## 4. Discussion

The choice for persons with disabilities to apply for support in parenting is not a decision taken lightly, and even when PA is granted, parents are very conscious about setting boundaries around their own role and responsibility as parents (Selander, 2015). In our sample, we found that the parents expressed a more modern meaning of being a parent. Fathers expressed caring tasks as their natural responsibility, and mothers talked about going out on leisure activities with their children [21].

Parents who use PA find that the support is important to be able to perform their parental duties, mostly in situations that require physical action, which has been described in previous studies [4,12,17]. PA has become scrutinized and the right has become increasingly circumscribed as showed in research [6] and very few parents receive support in their parenting [11]. This situation is expressed by parents who worry about losing their assistance as many respondents share the experience of getting less hours and even losing their assistance altogether. This has happened for some of the interviewed parents when they have felt a need for more assistance and have applied for more hours in parenting. None of the interviewed parents have said that their need for assistance has decreased, and no one has said that there are any improvements in their conditions that would lessen their needs for support. On the contrary, some parents say that having too little support in their lives causes harm to their health and has consequences for their children. Research on parenthood shows that socio-economic assets are important for the possibilities in how parents can exercise their parenthood [30]. Likewise for parents with disabilities, PA is a tangible asset in parenting.

Hence, decreasing assistance or losing assistance altogether has an impact both on parents and children. From a rights’ perspective and according to the CRPD, a person with a disability has a right to family life and to have the possibility to apply for support that can compensate parenting in situations where disability is experienced. As expressed by parents in this study as well as in previous research [17]. Disability in parenting occurs in *some* situations, but not in all areas of parenthood. Parents in the present study explain that PA in parenting is done strictly according to their rules.

From a rights’ perspective, there are direct consequences for children depending on how PA is given. There is impact on many aspects of their lives, for example, their possibilities to have activities in their leisure time, to get together and socialize with friends, and to safeguard their psychological well-being in feeling trust that their parent is taken care of. When PA is not provided or not sufficient, children are the ones present to support the parent, then taking on responsibilities as young carers [24]. The parents express criticism and reluctance to have to rely on their children for support. 

According to the CRC, children should have a voice in any decisions that influence their situation. Taken seriously, this would mean that in an assessment situation of the right for PA, the children of the family should be heard about what support they feel is needed for their parent in their everyday life. This is not, however, part of the current procedure of the assessment process. 

Also, in the CRC, children are granted the right to be cared for by their parents when possible. Hence, the construction of the LSS law must change, since parenthood when present should be regarded as a basic need; then the family life for the parent and the children should be assessed in terms of support for both parent and children. To sum up the discussion, almost thirty years after the implementation of PA, it is high time firstly to honor and act according to the value of equal participation as the foundation of disability policy when assessing the right to PA. Secondly, the LSS law must implement a child perspective on this intervention. It is in the best interests of the child to grant parents with disabilities PA as a basic support in the part of their parenting where it is needed.

## 5. Conclusions

In conclusion of this study, parents’ experiences are that PA allows them to fulfill their parental roles. The restrictions in hours and rules for the PA, however, shape an inflexible service that has consequences for both how parents can act in their parenting role and for children who miss out on social occasions and activities, and furthermore, may have to take on responsibilities towards their parent. Finally, taking the rights of children as expressed in CRC seriously, would mean to add childrens’ perspectives of their lives in to the assessment of entitlement to PA for parents.

## Data Availability

Data is not available due to ethical principles securing the privacy of the participating subjects.
